# Computational Refinement of Functional Single Nucleotide Polymorphisms Associated with *ATM* Gene

**DOI:** 10.1371/journal.pone.0034573

**Published:** 2012-04-13

**Authors:** C. George Priya Doss, B. Rajith

**Affiliations:** Centre for Nanobiotechnology, Medical Biotechnology Division, School of Biosciences and Technology, Vellore Institute of Technology University, Vellore, Tamil Nadu, India; University of Utah, United States of America

## Abstract

**Background:**

Understanding and predicting molecular basis of disease is one of the major challenges in modern biology and medicine. SNPs associated with complex disorders can create, destroy, or modify protein coding sites. Single amino acid substitutions in the *ATM* gene are the most common forms of genetic variations that account for various forms of cancer. However, the extent to which SNPs interferes with the gene regulation and affects cancer susceptibility remains largely unknown.

**Principal findings:**

We analyzed the deleterious nsSNPs associated with *ATM* gene based on different computational methods. An integrative scoring system and sequence conservation of amino acid residues was adapted for a priori nsSNP analysis of variants associated with cancer. We further extended our approach on SNPs that could potentially influence protein Post Translational Modifications in *ATM* gene.

**Significance:**

In the lack of adequate prior reports on the possible deleterious effects of nsSNPs, we have systematically analyzed and characterized the functional variants in both coding and non coding region that can alter the expression and function of *ATM* gene. *In silico* characterization of nsSNPs affecting *ATM* gene function can aid in better understanding of genetic differences in disease susceptibility.

## Introduction

There has been much effort in current epidemiology, medicine and phamarcogenomics studies identifying the genetic variations involved in complex diseases [1]. In particular ‘Single Nucleotide Polymorphisms’ (SNPs) are single nucleotide substitution in the nucleotide sequence that occurs at a frequency of approximately every 100 to 300 base pairs [2]. SNPs have been extensively used in genome-wide association studies to find the genomic regions that are susceptible to diseases and phenotypic variations. Even though most of the 14.6 million validated human SNPs in the dbSNP database (Build 131) are likely nonfunctional, some can alter cellular responses leading to a variety of disruptions, thereby increasing susceptibility to diseases like cancer [3]. About 2% of the all known single nucleotide variants associated with various disorders are non-synonymous SNPs (nsSNPs) in protein-coding regions (SNPs that alter a single amino acid in a protein molecule). SNPs in non coding regions may also have an impact on gene splicing, transcription factor binding or non-coding RNA [4]. Thus, special emphasis was laid to study the functional impact of SNPs in the coding region.

**Figure 1:**
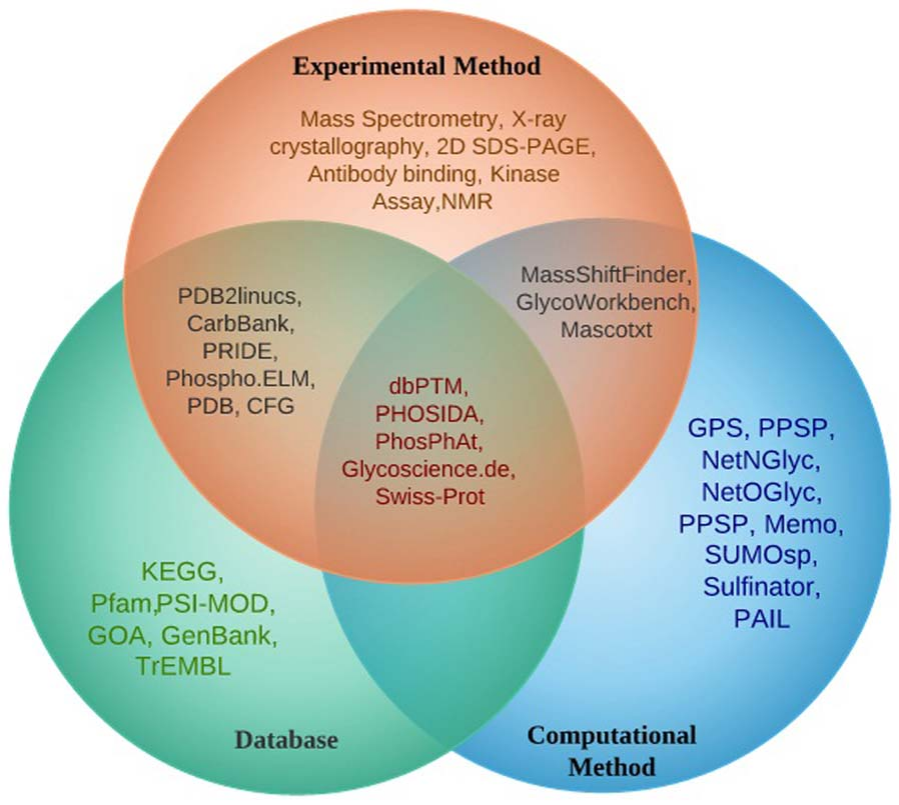
Flow chart for Post translational modification (PTM) analysis. Intersection between PTM related tools, databases and experimental determination techniques. *In silico* methods used for the analysis and storage of PTM annotations – set in the context of the experimental techniques that are used to detect them.

**Figure 2:**
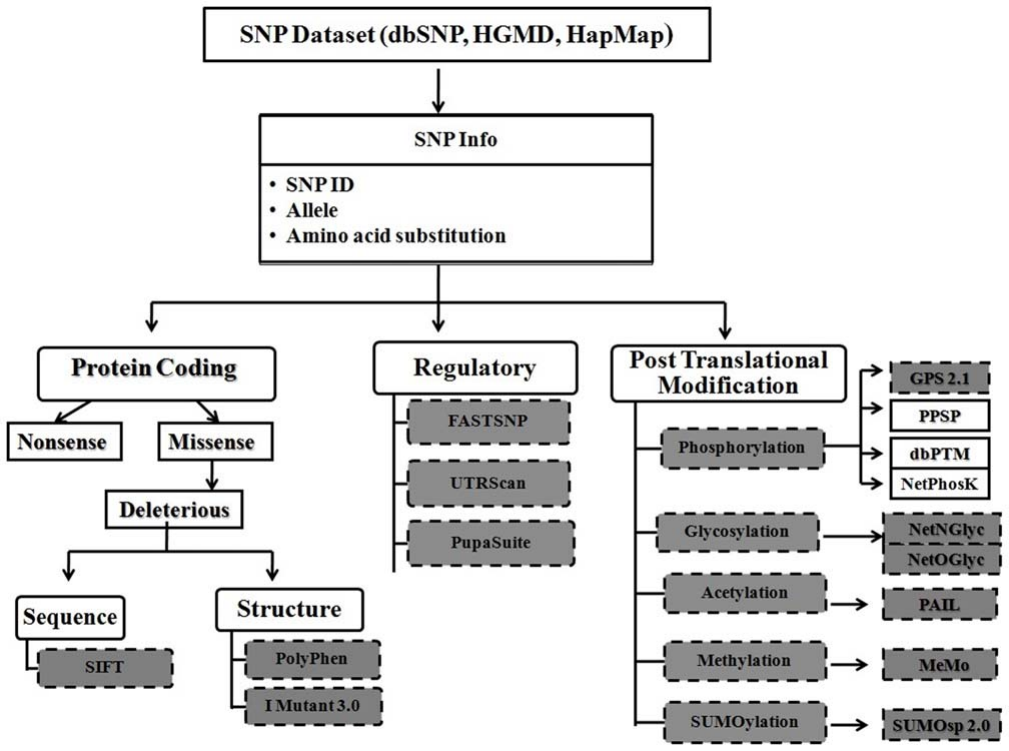
Schema representing the process of functional assessment of SNPs by *in silico* methods. SNPs were categorized based on its impact on coding region, regulatory region and post-translational modification sites. Once a tractable set of SNP is selected, *in silico* methods were used carefully to evaluate them based on the certain criteria specified by the users. Tools represented in shaded box were taken for our current analysis.

In recent years, there has been considerable interest in understanding the possible role of *ATM* gene in assessing the risk associated with cancer [5]–[27]. However, characterizing the point mutations associated with *ATM* at structural level is impossible and their results might not always reflect large scale *in vivo* genotype studies. In this context, to explore possible associations between genetic mutation and phenotypic variations in the absence of 3D structure, an evolutionary perspective to SNP screening was adopted using different algorithms like Sorting intolerant from tolerant (SIFT) [28], Polymorphism Phenotyping (PolyPhen) [29], I Mutant 3.0 [30], UTRScan [31], FastSNP [32] and PupaSuite [33]. However, these *in silico* methods provide arbitrary means of predicting the functional significance of SNPs with scores and annotation making the interpretation difficult. Deleterious SNPs in *ATM* gene and its impact on protein function have not been predicted so far using *in silico* methods. To address this issue, we have developed a scoring system that integrates the results from various *in silico* methods into a single coherent framework that enables better understating for experimental biologist. Disease causing mutation often resides in highly conserved positions. The evolutionary conservation analyses were calculated using the Bayesian method implemented in the ConSurf Web server (http://consurf.tau.ac.il) [34]. Further, the role of SNPs that could influence post-translational modification (PTM) of proteins was also studied. PTMs are implicated in many cellular processes and have a vital role in regulating the functional and structural property of proteins [35]. There are a number of reports which show the involvement of mutation in post-translational target sites leading to diseases [36]. The recent surge of interest in analyzing the PTMs has led to the development of several experimental methods to identify the PTMs on a genome wide scale [37]. Figure 1 displays the *in silico* resources that are commonly used for the analysis and storage of PTM annotations [38]–[42]. Even though a number of different PTMs are known protein Phosphorylation, Glycosylation and their respective analysis techniques have received more attention [43]–[45] than other modifications. Nevertheless, the role of PTMs such as protein Methylation, Acetylation and Sumoylation also remains significant in cellular function. Thus, we will rather discuss techniques for the prediction of some of these PTMs which are involved in causing a functional impact on *ATM* gene. Hence, our *in silico* study gains significance by (a) Predicting and prioritizing deleterious SNPs in the coding region using *in silico* approaches associated with *ATM* gene; (b) predicting the PTM sites related to *ATM* gene; and (c) validating our results by comparing them with experimentally proved data. A schema representing the process of functional assessment of SNPs using various *in silico* methods is illustrated in Figure 2. Our *in silico* analyses take advantage over experimental approach by its convenience, fast speed and low cost to locate the amino acids in the conserved region that regulate the function of ATM protein.

## Results

### SNP dataset

Polymorphism data of *ATM* gene investigated in this paper were retrieved from NCBI dbSNP database and Swiss Prot database. We selected non-synonymous and synonymous region SNPs from the coding region, untranslated (5′and 3′) and intronic region for our analysis.

### Prediction of deleterious nsSNPs in coding region

Among the 168 nsSNPs predicted in human *ATM* gene, 42 (25%), 117(69.6%) and 114(67.8%) were found to be deleterious, where as 126 (75%), 51 (30.4%) and 54 (32.2%) were found to be tolerated by SIFT, PolyPhen and I Mutant 3.0 (Table 1).

**Table 1:** The Prediction Results of nsSNPs of human *ATM* Using SIFT, PolyPhen and I Mutant 3.0 algorithms.

Prediction Result	SIFT		PolyPhen		I Mutant 3.0	
	No. of nsSNPs	%	No. of nsSNPs	%	No. of nsSNPs	%
**Deleterious**	42	25	117	69.6	114	67.8
**Tolerated**	126	75	51	30.4	54	32.2
**Total**	168	100	168	100	168	100

### Analysis of functional SNPs in the regulatory region

Functional SNPs in the regulatory region were analyzed using UTRScan, FastSNP and PupaSuite. UTR resource was applied to prioritize 49 mRNA UTR region. After comparing the functional elements SNP, we found that 11 SNPs in the 3′ UTR region and 2 SNPs in the 5′ UTR region were predicted to be functionally significant based on different functional pattern for each sequence (Table 2). FastSNP predicted 1 SNP at 5′ region with a risk ranking of (1–3) and 3 SNPs in intronic region having functional effect on the promoter/regulatory region with high risk ranking (3–4). PupaSuite helped in providing a platform for predicting the effect of SNPs on the structure and function of the affected protein. Among 168 SNPs, 34 nsSNPs were predicted to disrupt Exon Splicing Enhancer, 2 nsSNPs were predicted to disrupt Exon Splicing Silencer. PupaSuite predicted 15 SNPs in mRNA region and 1 SNPs in intronic region which disrupts Exon Splicing mechanisms (Table 2). A total of 36 nsSNPs (21.4%) and 16 mRNA SNPs (12.2%) were predicted to be functionally significant by PupaSuite.

**Table 2:** List of SNPs in regulatory region found to be functionally significant by PupaSuite, UTRScan and FASTSNP.

SNP	Region	PupaSuite	UTRScan	FASTSNP
**rs12284748**	mRNA	ESE	K-BOX	-
rs11558526	mRNA	ESE	-	-
**rs4987113**	mRNA	ESE	15-LOX- DICE, IRES	-
rs4987114	mRNA	ESE	-	-
rs3218711	mRNA	ESE	-	-
rs3092852	mRNA	ESE	-	-
**rs3092845**	mRNA	ESE	IRES	-
rs3092836	mRNA	ESS	-	-
rs3092834	mRNA	ESE	-	-
rs1137918	mRNA	ESE	-	-
rs453848	mRNA	ESE	-	-
rs378840	mRNA	ESE	-	-
**rs227092**	mRNA	ESE	IRES, K-BOX	-
**rs227091**	mRNA	ESE	15-LOX- DICE	-
rs189037	mRNA	ESE	-	-
**rs4585**	mRNA	ESE	15-LOX- DICE IRES	-
rs55900855	3′ UTR	-	IRES	-
rs4987114	3′ UTR	-	15-LOX- DICE, IRES	-
rs12284801	3′ UTR	-	IRES, K-BOX	-
rs4988000	3′ UTR	-	IRES	-
rs3218697	3′ UTR	-	IRES	-
rs3092844	3′ UTR	-	IRES	-
rs3092837	3′ UTR	-	K-BOX, IRES	-
rs4987880	5′ UTR	-	-	Promoter/regulatory region
rs4986839	Intron	-	-	splicing site
rs3092829	Intron	-	-	splicing site
rs3092872	Intron	-	-	splicing site

SNP IDs which are highlighted in bold were predicted to be functionally significant by PupaSuite and UTRScan.

ESE – Exon Splicing Enhancer, ESS- Exon Splicing Silencer.

### Concordance Analysis between SIFT and PolyPhen

A concordance study was performed to evaluate the prediction capacity of 168 nsSNPs predicted by SIFT and PolyPhen. The correlation analysis used raw scores rather than arbitrary defined categories. SIFT and PolyPhen scores showed a significant concordance between the predicted results (Spearman's ρ = −0.011; *P*≤0.02) as mentioned in Table 3.

**Table 3:** Concordance Analysis between the functional consequences of each nsSNP predicted by SIFT and PolyPhen.

PolyPhen	SIFT				
	Tolerated	Borderline	Potentially intolerant	Intolerant	Total
**Benign**	13	2	2	16	33
**Borderline**	3	0	0	6	9
**Potentially damaging**	8	2	6	8	24
**Possibly damaging**	20	4	6	31	61
**Probably damaging**	32	8	6	32	78
**Total**	76	16	20	93	205
Spearman's ρ = −0.011; *P*≤0.02					

PolyPhen- Benign (0.00–0.99); Borderline (1.00–1.24); Potentially damaging (1.25–1.49); Possibly damaging (1.50-1.99); Probably damaging (≥2.00).

SIFT-Tolerated (1.00–0.201); Borderline (0.20 - 0.101); Potentially intolerant (0.100 - 0.050); Intolerant (0.040-0.000).

### Integrative ranking system and of coding nsSNPs

We categorized SNPs predicted by various *in silico* methods based on highest annotated ranking scheme by which individual SNP could affect protein function. Based on our observation 20 nsSNPs (12%), 84 nsSNPs (50%), 52 nsSNPs (31.9%) and 12 nsSNPs (7.1%) were categorized under Rank I, II, III and IV respectively. Most deleterious SNPs were categorized under Rank I and the least significant SNPs were categorized in Rank IV (Table S1).

### Analysis of nsSNPs in conserved region

The ConSurf web server helped in identifying SNPs with variant position D140H, Y2677C, G2687R P2909G, G2687R and N3003D as highly conserved amino acid region as shown in Table 4.

**Table 4:** Conservation score of amino acid residues analyzed by Consurf.

nsSNPs	Amino acid	Conservation score	Function
VAR_010798	S49C	4	Exposed
VAR_041546	D140H	9	Highly conserved and exposed
rs35963548	C532Y	5	Buried
VAR_041557	P872S	1	Exposed
VAR_056683	L942F	5	Buried
rs12788429	V1161G	7	Buried
rs35962982	L1590F	3	Buried
VAR_056688	R2034Q	6	Buried
VAR_010853	E2423G	8	Exposed
VAR_010854	V2424G	8	Exposed
VAR_010856	T2438I	8	Exposed
VAR_056690	E2570G	2	Exposed
VAR_010863	D2625Q	7	Exposed
rs28942103	Y2677C	9	Highly conserved and exposed
VAR_041582	P2842R	7	Buried
VAR_010886	G2867R	9	Highly conserved and buried
rs56887719	P2907L	9	Highly conserved and exposed
VAR_010890	E2909G	9	Highly conserved and exposed
rs1137889	N3003D	8	Highly conserved and exposed

Conservation Score: 1–4 Variable; 5–6 Intermediate; 7–9 Conserved.

### Prediction of Post-translational modification sites

**Figure 3:**
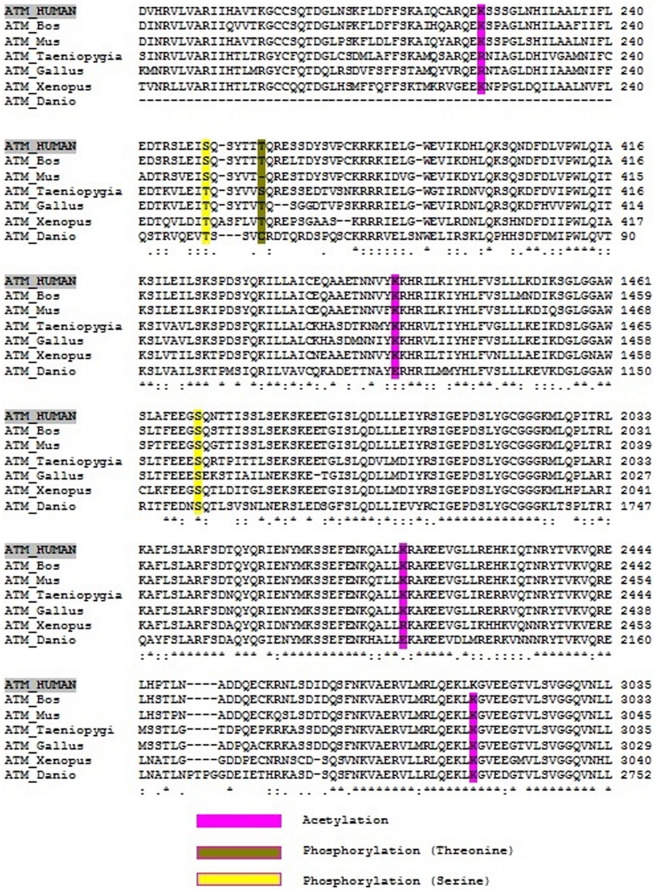
Summary of the multiple sequence alignment of different vertebrate sequences for PTM sites. Human *ATM* gene were compared with four different species. i) Mammals- *Mus musculus* (EDL25796.1) and *Bos Taurus* (NP_001192864.1), ii) Amphibia - *Xenopus tropicalis* (NP_001081968.1), iii) Aves - *Taeniopygia guttata* (XP_002197770.1) and Gallus gallus (NP_001155872.1), iv) Actinopterygii - *Danio rerio* (BAD91491.1). The consensus sequence is marked by an asterisk, conserved substitution by a double dot, and semi conserved substitution by a single dot. The different sequences are ordered as in aligned results from ClustalW.

In this study, we have used *in silico* approaches for the prediction of various post translational modifications associated with *ATM* gene. 11 serine specific Phosphorylation sites and 1 tyrosine specific site were predicted by Group-based Phosphorylation Scoring method (GPS 2.1). Nearly all the phosphorylated sites predicted by GPS 2.1 were found to be conserved across the species as mentioned in Figure 3. Glycosylation is another type of PTMs and which is implicated in protein folding, transport and function. NetNGlyc 1.0 server predicted 9 glycosylation sites at 81, 272, 704, 765, 789, 1230, 1240, 1719, and 1983 positions and NetOGlyc 3.1 server predicted 2 glycosylation sites at 2666 and 2902 positions respectively. MeMo is a web based protein methylation modification prediction tool. According to MeMo, 20 methylation sites were predicted in which, 4 were arginine specific and 16 were Lysine specific methylation sites as shown in Table 5. Protein acetylation sites were predicted using Prediction of Acetylation on Internal Lysines (PAIL), which could predict 86 potential lysine acetylation sites in human as shown in Table 5. Protein Sumoylation is important reversible PTMs and orchestrates a variety of cellular processes. Our analysis revealed the presence of six type II non consensus sites and four type I lysine sites using SUMOsp 2.0.

**Table 5:** Prediction of various PTM residues with its positions using different *In silico* tools.

Phosphorylation	Methylation	Glucosylation		SUMOylation	Acetylation	
GPS 2.1	Memo	NetNGlyc	NetOGlyc	SUMOsp 2.0	PAIL	
**Serine**	**Arginine**	**Asparagine**	**Threonine**	**Lysine**	**Lysine**	
**367**	329	81	2666	24	24	1582
403	493	272	2902	477	25	1615
475	832	704		640	29	1665
875	2719	765		892	31	1692
1360	2811	789		1323	41	1701
1635	**Lysine**	1230		2153	50	1738
**1981**	331	1240		2302	53	1772
2242	573	1719		2421	79	1773
2592	1066	1983		2456	93	1782
2761	1109			2687	102	1820
2941	1196				106	1834
**Threonine**	1330				116	1903
**373**	1772				**224**	1964
	1992				296	1965
	2266				300	1992
	2331				331	1994
	2418				342	2025
	2585				385	2117
	2700				387	2148
					388	2213
					397	2303
					468	2331
					556	2383
					640	2385
					687	**2418**
					750	2421
					792	2440
					793	2456
					797	2460
					926	2585
					1109	2589
					1170	2639
					1178	2687
					1181	2710
					1192	2717
					1280	2747
					1398	2789
					1400	2804
					**1435**	2848
					1454	2992
					1510	**3018**

Amino acid positions highlighted in bold were found to be experimentally proved.

## Discussion

Since the functional impact of most SNPs remains unknown, choosing target SNPs for an investigation continues to be an unresolved issue. To address this issue, we used different *in silico* methods based on the combination of two diverse approaches namely sequence and structural based approaches. Sequence-based prediction methods are one step ahead of the structure-based methods, as they can be applied to any proteins with known relatives. In contrast, structure based approaches have limited application, as they are not feasible to implement for proteins with unknown 3D structures. Tools that integrate both sequence and structure resources have added advantage of being able to assess the reliability of the prediction results by cross-referencing the results from both approaches. Most *in silico* methods utilizes this information for the prediction of deleterious nsSNPs, among which SIFT and PolyPhen algorithm are the main representatives. Defining that the variants whose positions with normalized probability score<0.05 in SIFT and a PSIC score>1.5 in PolyPhen are predicted to be deleterious, 25% and 69.6% of amino acid substitution were predicted to have functional impact on *ATM* gene. The variation in prediction score of SIFT and PolyPhen is mainly due to the difference in protein sequence alignment and the scores used to classify the variants [46]. Significant concordance was observed between the functional consequences of nsSNP predicted by SIFT and PolyPhen (Spearman's ρ = −0.011; *P*≤0.02). Recent analysis by Flanagan et al. [47] have confirmed the accuracy of SIFT and PolyPhen in predicting the effect of nsSNPs on protein function. In order to validate and substantiate the prediction accuracy of SIFT and PolyPhen, our results were compared with experimentally proved study. It has been estimated that 61.9% and 67% of nsSNPs were correctly predicted as deleterious by SIFT and PolyPhen (Table 6). In addition, the Pearson χ^2^ test shows that prediction scores of SIFT and PolyPhen were in significant correlation with the numbers of nsSNPs with known phenotype (Table 6). In order to predict the impact of nsSNPs on protein structure, I Mutant 3.0 was used which evaluate the stability change upon single site mutation. I Mutant 3.0 was ranked as one of the most reliable predictor based on the work performed by Khan and Vihinen [48]. Based on the difference in Gibbs free energy value of mutated and wild type protein, 67.8% nsSNPs were found to largely destabilizes the protein (<−0.5 Kcal/mol) out of which 88.9% nsSNPs are experimentally validated.

**Table 6:** Correlation analysis between prediction score for deleterious and validated nsSNPs by *In Vivo/In Vitro* analysis.

Algorithm	Category	Number of deleterious nsSNPs	nsSNPs validated by *In Vivo/In Vitro* analysis
**SIFT** *P* value = 0.60	0	15	9
	0∼0.05	37	17
	Total	42	26
**PolyPhen** *P* value = 0.048	Probably damaging	64	49
	Possibly damaging	53	30
	Total	117	79

The ranking system that we developed to assess the *in silico* information is intended for use in the absence of biochemical characterization and 3D structure information. Such an integrative scoring system will aid in prioritizing functional nsSNPs and further experimental analysis may strengthen our analysis. Highly ranked nsSNPs (Rank I) on account of deleterious nature were further selected for the quantification of conserved residues. Population genetic analysis indicates that a significant fraction of functional nsSNPs were present in the conserved region. Doniger et al. and Aly et al. validated the role of functional SNPs within evolutionary conserved regions [49]–[50]. Hence nsSNPs at position D140H, Y2677C, G2867R, P2907L, E2909G and N3003D present in the highly conserved region were found to be most deleterious and predicted to have functional impact on ATM protein [5], [7], [23]. Currently more interest has been focused on functional SNPs affecting regulatory regions or the splicing process. In this context, we used PupaSuite to pin-point the exact effect of a mutation to a specific structural or physicochemical property, ranging from disruption of protein-protein interactions to protein aggregation. Further, we examined what kind of bio-molecular property SNPs mainly affects. Recall the SNPs in exonic regions that may affect protein coding, PTM or splicing regulation we compare our system with UTRScan and FastSNP that predict the deleterious affect of SNPs. FastSNP server could not predict the functional impact of SNPs in the 3′ region. The functional pattern change predicted by UTRScan includes IRES, 15-LOX- DICE and K-BOX. IRES are bound by internal mRNA ribosome. IRES are involved in internal mRNA ribosome binding system which controls the translational mechanisms in cell cycle [51]. 15-lipoxygenase differentiation control element (15-LOX-DICE) controls 15-LOX synthesis which catalyses the degradation of lipids and mitochondrial products during reticulocyte maturation. The following SNPs with IDs rs12284748, rs4987113, rs4987114, rs3092845, rs227092, rs227091 and rs4585 were found to have functional significance by both UTRScan and PupaSuite.

PTM of proteins provides reversible means to regulate different function of proteins and is implicated in almost all cellular processes. More than 32 *in silico* methods for PTM sites prediction were developed based on different requirements [52]. Among this protein phosphorylation being one of the most-studied one, we employed GPS 2.1 for the prediction. The two serine residues at positions 367, 1981 and one tyrosine residue at 373 positions predicted by GPS 2.1 were validated by experimental studies [53], [54]. Phosphorylation of Ser 1981 is the most extensively studied phosphorylation in human *ATM* [55]. Both the potential phosphorylated serine residues at 367 and 1981 positions were also found to be conserved in mammals, amphibians, birds, and actinopterygii, except for threonine 373 with a deletion gap (DG) in the Mus Muculus as shown in Figure 3. Hence the mutations which create phosphorylation sites destabilize proteins, interrupt protein interactions or disrupt normal protein functions. They may also recruit kinases or phosphatases necessary for other cellular processes causing system-wide deregulation. Protein acetylation is also an important PTM which regulates much cellular activity. Hence PAIL was used for the acetylation site prediction. The accuracy of PAIL among other prediction tool have been confirmed using both Jack-Knife and cross validation method [41]. Out of 87 potential acetylation site predicted by PAIL, 4 lysine sites at positions 224, 1435, 2418 and 3018 were in concordance with the experimental results [11], [18], [55]–[56]. All four lysine site were found to be conserved in mammals, amphibians, birds, and Actinopterygii as shown in Figure 3. PTM sites involved in Acetylation (K3018N, K1454N) and Glycosylation (N1983S) were predicted to be functionally significant by the *in silico* analysis and have been validated with experimental support [7], [17], [21]. However, more detailed experimental studies are required to validate the role of predicted SNPs in PTM sites. Using these *in silico* approaches, precise and useful information about the effects of mutations on protein structure and function can be readily obtained. Some of our earlier studies have helped in predicting the functional nsSNPs associated with cancer related genes such as *TP53*, *HNPCC*, *ARNT* and *BRCA1*. Our findings revealed that analysis which employs sequence and structure based approaches as a pipeline in prioritizing candidate functional nsSNPs [57]–[61]. In addition, we validated impact of predicted deleterious nsSNPs at structural level based on RMSD, ASA, and DSSP analysis. Since the 3D structure of ATM protein is not available in protein data bank, we proposed an alternative method for characterizing the functional and deleterious SNPs using integrative ranking in combination with conservation analysis. To summarize with, the goal of the current study was to integrate relevant biomedical information sources to provide a novel approach for cancer associated gene. Our study gains significance by predicting the possible deleterious SNPs and the PTM sites associated with *ATM* gene, so that the number of SNPs screened in association with diseases can be narrow down to those that are most likely to alter gene function. We anticipate that the results obtained from our analysis would pave a way by providing useful information to the researchers and can play an important role in bridging the gap between biologists and bioinformaticists.

## Materials and Methods

### Retrieval of SNPs

The SNP information of *ATM* gene was retrieved from the NCBI dbSNP (http://www.ncbi.nlm.nih.gov/snp/), HGMD database (http://www.hgmd.org/) and the HapMap database (http://hapmap.ncbi.nlm.nih.gov/) for our studies. The information on the impact of the nsSNP variation and its association with disease were compiled from *in vivo* and *in vitro* experiments according to Pub Med (http://www.ncbi.nlm.nih.gov/PubMed/), OMIM (http://www.ncbi.nlm.nih.gov/omim/), and UniProtKB (http://www.uniprot.org/).

### Functional Prediction of Amino Acid-Substitution Variations in protein coding region

There are several online tools which employ sequenced based approaches for the prediction of nsSNPs. We used the most recent version SIFT (http://blocks.fhcrc.org/sift/SIFT.html) BLink Beta for our studies. We submitted query in the form of gene identification number (GI) obtained from NCBI database. SIFT score implies the tolerance index of a particular amino acid substitution that alters the protein function. SIFT calculates the probability that an amino acid change at a particular position is tolerated. Output scores are in the range from 0 to 1, with 0 being damaging and 1 being neutral [62]. If any of the scores are lower than the cutoff of 0.05 used by SIFT, the respective amino acid substitution would then be predicted to be deleterious. The alignment built by SIFT algorithm contains homologous sequences with a medium conservation measure of 3.0 [63] to minimize false positive and false negative error. The output of SIFT is a table of probabilities for each amino acid at each position as well as predictions on not tolerated or tolerated amino acids for each position. The accuracy of the SIFT is validated by experimental proteins analyzed by various groups Cargill et al. [64], Palmer et al [65]. PolyPhen (http://genetics.bwh.harvard.edu/pph/) predicts the possible impact of amino acid substitutions on proetin structure and function using straight forward physical and evolutionary comparative considerations. The input of PolyPhen is an amino acid sequence or corresponding ID with the position of the amino acid variant. PolyPhen searches for 3-D protein structures, multiple alignments of homologous sequences and amino acid contact information in several protein structure databases. Then it calculates PSIC scores for each of two variants, and computes the difference of the PSIC scores of these variants. The higher a PSIC score difference the higher is the functional impact a particular amino acid substitution is likely to have. A PSIC score difference of 1.5 and above is considered to be damaging. The PolyPhen scores can be classified as probably damaging (≥2.00), possibly damaging (1.50–1.99), potentially damaging (1.25–1.49), or benign (0.00–0.99). This enables quantitative ranking of the severity of the effects of SNPs on resulting protein phenotypes, leading to the prioritization of the most biologically significant SNPs for experimental studies. I-Mutant 3.0 (http://gpcr2.biocomp.unibo.it/cgi/predictors/I-Mutant3.0/I-Mutant3.0.cgi) is a support vector machine (SVM)-based tool. Input for I-Mutant 3.0 is either a protein structure or a sequence. We used the sequence-based version of I Mutant3.0 which classifies the prediction in three classes: neutral mutation (−0.5≤DDG≤0.5 Kcal/mol), large Decrease (<−0.5 Kcal/mol) and large Increase (>0.5 Kcal/mol). The output file shows the predicted free energy change (DDG) which is calculated from the unfolding Gibbs free energy change of the mutated protein minus the unfolding free energy value of the native protein (Kcal/mol).

### Defining the Functional context of SNPs in the regulatory region

The functional impacts of SNPs in regulatory regions were analyzed using UTRScan, FastSNP and PupaSuite. UTRScan was used for the analysis of SNPs in the regulatory untranslated region. 5′UTR and 3′UTR of eukaryotic mRNAs are involved in many post transcriptional regulatory pathways that control mRNA localization, stability and translation efficiency [66]. UTRScan looks for UTR functional elements by searching through user submitted query sequences for the patterns defined in the UTRsite collection. UTRsite is a collection of functional sequence patterns located in 5′ or 3′UTR sequences. If different sequences for each UTR SNP are found to have different functional patterns, then it is predicted to have functional significance. The internet resources for UTR analysis (http://itbtools.ba.itb.cnr.it/utrscan) were UTRdb and UTRsite. UTRdb contains experimentally proven biological activity of functional patterns of UTR sequence from eukaryotic mRNAs. FastSNP identifies the polymorphism involving the intron which may lead to defects in RNA and mRNA processing. It is an integrated platform application that analyzes a known polymorphism in a given gene or list of genes offering great benefits to the user in terms of speed and convenience. The FastSNP server (http://fastsnp.ibms.sinica.edu.tw) follows the decision tree principle with external Web service access to TFSearch, which predicts whether a non-coding SNP alters the transcription factor binding site of a gene. The score will be given on the basis of levels of risk with a ranking of 0, 1, 2, 3, 4, or 5. This signifies the levels of no, very low, low, medium, high, and the very high effect, respectively. FastSNP tool helped in classifying and prioritizing deleterious effects of SNPs based upon their influence over determining protein structure, pre-mRNA splicing, deviation in transcriptional levels of the sequence, alterations in the premature translation termination, deviations in the sites at promoter region for transcription factor binding etc. SNPs were analyzed using PupaSuite to identify those with putative deleterious functional impact designations like determining whether they are located in possible conserved regions, transcription factor binding sites, exonic splice enhancer sites, exonic splice silencer sites, triplet formation sites or intron-exon boundaries. PupaSuite combines the functionality of PupaSNP and PupasView in a unique and more integrated interface, and adds new modules to facilitate the selection of the optimal set of SNPs for a large-scale genotyping studies.

### Ranking SNPs based on Integrative scoring system

**Figure 4:**
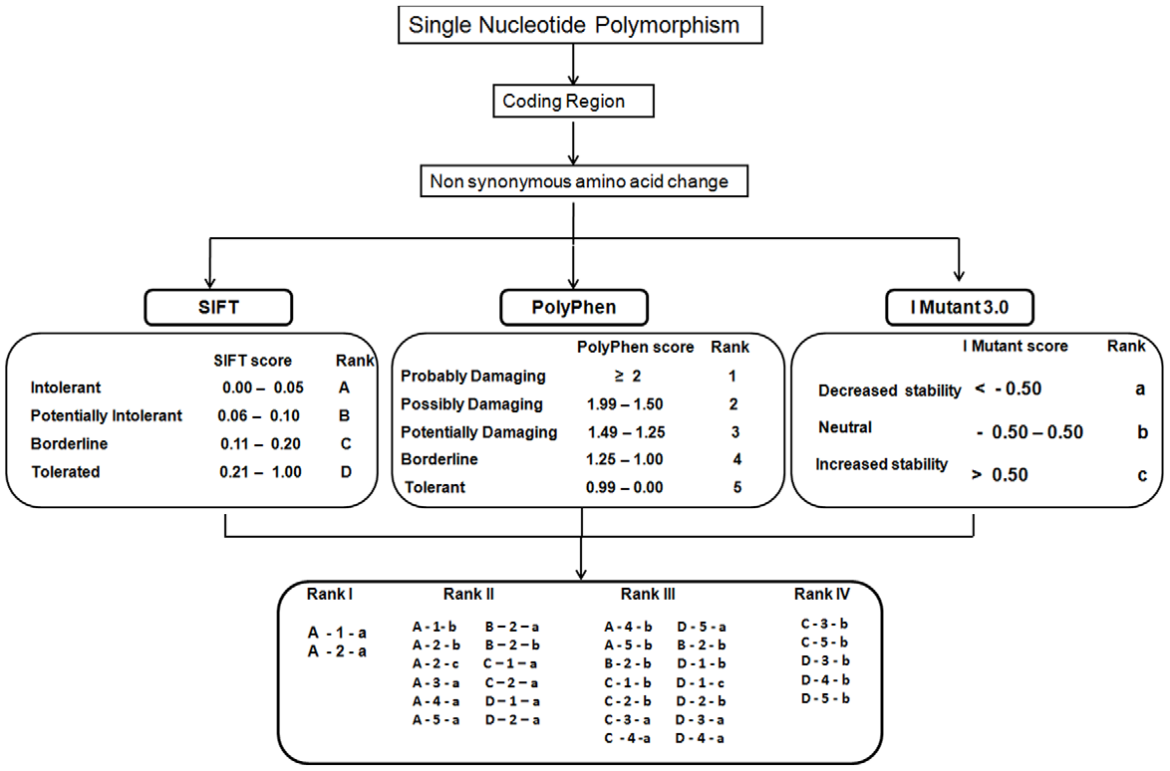
Integrative ranking system for nsSNPs in coding region. Predicted SNPs were categorized into four ranking groups based on the degree of deleterious effects. Coding SNPs were evaluated based on scores from SIFT, PolyPhen and I Mutant 3.0.

We considered multiple ways a SNP could have impact on protein function. SNPs with the highest likelihood of being functionally relevant and therefore most important to interrogate were ranked based on SIFT, PolyPhen and I Mutant 3.0 scores (Figure 4). Rank I SNPs have the most potential for functional significance and Rank IV SNPs have the least potential for functional significance.

### Detection of potential PTM sites in *ATM* gene

Many approaches have been proposed for PTM site recognition. Some methods, such as identifying physicochemical properties and searching motif patterns, have been developed and applied to the prediction of PTM sites. Some of the bioinformatics resources that have been created to aid in the analysis and storage of PTM annotations are listed in Table 1. Among them, GPS 2.1 was used for our analysis. Medium level threshold with a cutoff value 4.16 was chosen for the identification of phosphorylation site. Another ubiquitous PTM is protein glycosylation that occur as N-glycosylation and O-glycosylation. NetNGlyc was used to predict N-glycosylation and NetOGlyc 3.1 was used to predict O-glycosylation respectively. NetNGlyc 1.0 server predicts N-Glycosylation sites in human proteins using artificial neural networks that examine the sequence context of Asn-Xaa-Ser/Thr sequences. The NetOGlyc server produces neural network based predictions of mucin type GalNAc O-glycosylation sites in mammalian proteins. The G-score is the score from the best general predictor; the I-score is the score from the best isolated site predictor. If the G-score is >0.5, the residue is predicted as glycosylated; the higher the score more confident the prediction. Protein methylation can modify the nitrogen atoms of either the backbone or side-chain (N-methylation) of protein [67]. In this study, we used Memo which is a novel tool for predicting protein methylation function and dynamics. MeMo predicts methylation of arginine and lysine via SVMs strategy. Protein acetylation is an essentially reversible post-translational modification which regulates diverse protein properties such as protein-protein interaction, DNA binding, enzymatic activity, stability and sub cellular localization [68]. PAIL is a novel predictor tool for the identification of protein acetylation sites with great accuracy. Sumoylation involves the covalent attachment of small ubiquitin-like modifier (SUMO) peptide to lysine side chain in acceptor proteins which results in altered protein activity and stability. The prediction of Sumoylation was done using SUMOsp 2.0 server. Medium level threshold with a cutoff value 2.64 was chosen for the identification of Sumoylation sites.

### Sequence analysis for highly conserved variants

Amino acid sequence of *ATM* protein was retrieved from Swiss-Prot. BLAST (Basic Local Alignment and search tool) available in NCBI database (http://blast.ncbi.nlm.nih.gov/) was used for retrieving a similar sequence to the target *ATM* protein. The conservation scores of amino acid variant were calculated using ConSurf web server. It calculates the evolutionary conservation of amino acid positions in proteins using an empirical Bayesian inference. Highly conserved amino acids from proteins were used for further analysis.

### Statistical Analysis

Spearman's rank correlation **c**oefficient ρ was used for analyzing Concordance between the functional consequences of each nsSNP of *ATM* genes predicted by the two *in silico*. We used Pearson's χ^2^ test [69] to study the correlation analysis between functionally significant predicated nsSNPs and nsSNPs validated by *in vivo/in vitro* analysis, in which values below 0.05 were considered statistically significant.

## Supporting Information

Table S1 **Summary of nsSNPs that were prioritized by SIFT, PolyPhen, I Mutant 3.0 and PupaSuite.**
(DOC)
