# Supplementary material for: Computational Refinement of Functional Single Nucleotide Polymorphisms Associated with ATM Gene
Source: PLoS One. 2012 Apr 13;7(4):e34573. doi: 10.1371/journal.pone.0034573 (PMC3326031; doi:10.1371/journal.pone.0034573)
Supplement: Table S1 — Summary of nsSNPs that were prioritized by SIFT, PolyPhen, I Mutant 3.0 and PupaSuite. (DOC) [file pone.0034573.s001.doc]

**Table S1. Summary of nsSNPs that were analyzed by SIFT, PolyPhen, I Mutant 3.0 and PupaSuite in *ATM* gene**

**NA-Not available; SNP IDs which are highlighted in bold were predicted to be functionally significant by SIFT/ PolyPhen/ I Mutant 3.0. SNPs are ranked based on the degrees of functional impact (Rank I – most deleterious, Rank IY – tolerated) ESE – Exon Splicing Enhancer, ESS- Exon Splicing Silencer**

| **Reference sequence IDs** | **Allele** | **SIFT** | **PolyPhen** | **I Mutant**  **3.0** | **Rank** | **Pupasuite** | **Reference** |
| --- | --- | --- | --- | --- | --- | --- | --- |
| rs7112053 | L3V | 0.18 | 1.21 | -1.31 | III | ESE |  |
| VAR_056678 | R45W | 0.18 | 1.996 | -0.30 | III | -NA- |  |
| **VAR_010798** | **S49C** | **0.00** | **1.662** | **-0.57** | **I** | -NA- | [5] |
| rs35389822 | I68V | 0.00 | 0.141 | -0.94 | II | -NA- |  |
| rs1442730 | R114K | 0.19 | 1.37 | -0.83 | III | ESE |  |
| VAR_010799 | D126E | 0.05 | 1.22 | -0.3 | II | -NA- | [6 ] |
| rs2234998 | C134R | 0.25 | 1.572 | -0.7 | II | -NA- |  |
| **VAR_041546** | **D140H** | **0.00** | **2.064** | **-0.75** | **I** |  | [7] |
| rs35858242 | S160F | 0.02 | 1.781 | 0.52 | II | ESE |  |
| VAR_010800 | V182L | 0.19 | 0.672 | -1.21 | III |  | [8] |
| rs2235002 | A216S | 0.14 | 0.939 | -0.65 | III | -NA- |  |
| VAR_010801 | K223E | 1.00 | 1.344 | -0.29 | III |  | [9] |
| VAR_041547 | R250Q | 0.25 | 1.595 | -1.1 | II |  | [7] |
| rs35261362 | I289T | 0.76 | 1.643 | -1.73 | II | ESE |  |
| VAR_010802 | P292L | 0.10 | 2.724 | 0.02 | III |  | [10] |
| VAR_010803 | I323V | 0.02 | 0.96 | -1.14 | II |  | [11] |
| VAR_010804 | Y332C | 0.73 | 2.616 | -1.32 | II |  | [12 |
| VAR_041548 | S333F | 0.14 | 1.893 | -0.33 | III |  | [7] |
| VAR_041549 | R337C | 0.68 | 2.495 | -1.39 | II |  | [7] |
| VAR_041550 | R337H | 0.76 | 1.82 | -1.72 | II |  | [7] |
| VAR_010805 | A350T | 0.03 | 1.413 | -0.78 | II |  | [13] |
| VAR_010806 | I352T | 0.58 | 1.758 | -2.18 | II |  | [13] |
| rs34083085 | Y380N | 0.58 | 1.955 | -1.47 | II | -NA- |  |
| VAR_041551 | V410A | 0.03 | 1.467 | -0.9 | II |  | [7] |
| VAR_041552 | N504S | 0.54 | 0.521 | 0.03 | IV |  | [7] |
| VAR_010807 | C514D | 0.59 | 1.49 | -0.92 | III |  | [7], [9] |
| **rs35963548** | **C532Y** | **0.03** | **3.084** | **-0.5** | **I** | ESE |  |
| VAR_041553 | C540Y | 0.03 | 2.328 | -0.01 | II |  | [7] |
| VAR_041554 | L546V | 0.49 | 1.151 | -1.02 | III |  | [7] |
| VAR_010808 | F570S | 0.78 | 1.43 | -1.76 | III |  | [5] |
| VAR_041555 | F582L | 0.30 | 0.71 | -1.08 | III |  | [7] |
| rs2227922 | P604S | 0.33 | 0.955 | -1.45 | III | -NA- |  |
| VAR_010810 | S707P | 0.33 | 0.409 | -0.23 | IV |  | [8] |
| rs55830714 | R720H | 0.02 | 0.00 | -1.48 | II | -NA- |  |
| rs3205809 | Q754K | 1.00 | 1.771 | -0.25 | III | -NA- |  |
| VAR_056679 | T761S | 0.31 | 0.364 | -0.62 | III |  |  |
| rs34231402 | F763L | 1.00 | 0.771 | -1.02 | III | -NA- |  |
| VAR_010812 | N768D | 0.02 | 1.503 | -0.29 | II |  | [10] |
| VAR_010813 | R785C | 1.00 | 0.44 | -0.76 | III |  | [5] |
| VAR_056680 | S788R | 0.95 | 0.093 | 0.02 | IV |  |  |
| VAR_056681 | D814E | 1.00 | 1.389 | -0.22 | III |  |  |
| VAR_041556 | E848Q | 0.36 | 0.197 | -0.66 | III |  | [7] |
| VAR_010814 | F858L | 0.14 | 2.022 | -1.34 | II |  | [14] |
| **VAR_041557** | **P872S** | **0.02** | **1.552** | **-1.5** | **I** |  | [7] |
| rs3205810 | E887G | 0.24 | 1.865 | -1.02 | II | -NA- |  |
| VAR_041558 | R924W | 0.90 | 2.359 | -0.55 | II |  | [6] |
| rs3218708 | T935M | 0.22 | 0.636 | -.0.06 | IV | ESE |  |
| VAR_041559 | T935A | 0.24 | 0.636 | -.0.06 | IV |  | [7] |
| VAR_056682 | T935M | 0.01 | 0.918 | -0.78 | II |  |  |
| **VAR_056683** | **L942F** | **0.02** | **1.568** | **-0.73** | **I** |  |  |
| VAR_010815 | L942R | 0.22 | 2.018 | -1.17 | II |  |  |
| VAR_010816 | L1001Q | 0.92 | 2.018 | -2.02 | II |  | [10] |
| VAR_010817 | M1040V | 0.05 | 0.784 | -1.09 | II |  | [15] |
| VAR_010818 | P1054R | 0.22 | 2.621 | -0.94 | II |  | [7] |
| VAR_010819 | H1082L | 1.00 | 3.371 | 0.84 | III |  |  |
| VAR_010820 | E1091D | 0.01 | 1.036 | -0.46 | III |  | [16] |
| rs56398245 | Q1128R | 0.61 | 1.74 | -0.07 | III | -NA- |  |
| rs12788418 | V1153G | 0.29 | 1.546 | -2.62 | II | ESE |  |
| rs12788427 | V1160G | 0.38 | 1.648 | -2.37 | II | -NA- |  |
| **rs12788429** | **V1161G** | **0.01** | **2.264** | **-2.37** | **I** | ESE |  |
| VAR_041560 | S1179F | 0.61 | 2.149 | 0.54 | III |  | [7] |
| rs12786957 | E1186D | 0.59 | 0.098 | -0.39 | IV | ESE |  |
| rs12786960 | H1188P | 0.20 | 2.513 | 0.37 | III | ESE |  |
| VAR_056684 | E1313Q | 0.29 | 1.384 | -0.44 | IV |  |  |
| VAR_041561 | M1321I | 0.38 | 0.515 | -0.55 | III |  | [7] |
| VAR_041562 | H1380Y | 0.01 | 0.32 | 0.11 | III |  | [7] |
| VAR_041563 | P1382S | 0.59 | 0.634 | -1.36 | III |  | [7] |
| VAR_010821 | I1407T | 0.20 | 1.758 | -1.84 | II |  | [15] |
| VAR_010822 | L1420F | 0.53 | 1.444 | -0.89 | III |  | [7] |
| VAR_010823 | L1420P | 0.24 | 1.969 | -0.89 | II |  | [11] |
| VAR_056685 | A1427T | 1.00 | 0.216 | -0.62 | III |  |  |
| VAR_010824 | K1454N | 0.48 | 1.552 | -0.36 | IV |  | [17] |
| VAR_010825 | F1463S | 0.17 | 2.603 | -1.85 | II |  | [15] |
| VAR_010826 | L1465P | 0.60 | 2.243 | -1.7 | II |  | [8] |
| VAR_041564 | I1469M | 0.15 | 1.424 | -1.73 | III |  | [7] |
| VAR_041565 | Y1475C | 0.25 | 2.384 | -1.2 | II |  | [7 |
| VAR_056686 | L1541 | 0.00 | 1.568 | -0.84 | I |  |  |
| VAR_010827 | P1566R | 0.24 | 2.621 | -0.98 | II |  | [16] |
| VAR_010828 | V1570A | 0.28 | 0.432 | -1.71 | III |  | [7] |
| **rs35962982** | **L1590F** | **0.00** | **1.568** | **-1.3** | **I** | ESE |  |
| rs681518 | Q1620P | 0.24 | 2.318 | -0.44 | III | -NA- |  |
| rs56354559 | H1624R | 0.28 | 2.461 | -0.11 | III | -NA- |  |
| rs55843558 | M1644T | 0.07 | 2.076 | -0.82 | II | -NA- |  |
| VAR_041566 | N1650S | 0.07 | 0.093 | -0.29 | IV |  | [7] |
| VAR_010829 | 1682H | 0.93 | 2.189 | -0.5 | III |  | [15] |
| VAR_010830 | S1691 | 0.49 | 1.205 | 0.05 | IV |  | [10] |
| VAR_056687 | V1729L | 0.37 | 1.208 | -1.15 | III | ESS |  |
| VAR_041567 | N1739T | 0.27 | 1.733 | 0.25 | III |  | [7] |
| VAR_010831 | T1743I | 0.08 | 1.818 | -0.32 | III |  | [10] |
| rs35556390 | Q1765P | 0.27 | 2.131 | -0.01 | III | ESE |  |
| VAR_010833 | D1853N | 0.05 | 2.639 | 0.1 | II |  | [7] |
| VAR_010834 | D1853C | 0.10 | 1.739 | -2.76 | II |  | [7 |
| VAR_010835 | L1910H | 0.46 | 2.243 | -2.37 | II |  | [15] |
| VAR_010836 | V1913G | 0.81 | 1.548 | -2.18 | II |  |  |
| VAR_041568 | M1916I | 0.42 | 1.363 | -0.78 | III |  | [7] |
| VAR_041569 | A1945T | 0.59 | 1.327 | -0.67 | III |  | [7] |
| VAR_010837 | T1953R | 0.40 | 2.05 | -0.18 | III |  | [18] |
| VAR_041570 | Y1961C | 0.66 | 2.758 | -0.84 | II |  | [7] |
| VAR_041571 | N1983S | 0.17 | 0.263 | -0.14 | IV |  | [7] |
| VAR_041572 | E1991D | 0.15 | 1.347 | -0.37 | IV |  | [7] |
| VAR_010838 | D2016G | 0.80 | 2.189 | -1.23 | II |  | [5] |
| rs35991214 | G2022S | 0.81 | 1.824 | -0.91 | II | ESE |  |
| rs11212587 | G2023R | 0.42 | 2.274 | -0.23 | III | ESS |  |
| **VAR_056688** | **R2034Q** | **0.05** | **1.754** | **-0.64** | **I** |  |  |
| VAR_010839 | G2063E | 0.31 | 2.274 | -0.44 | III |  |  |
| VAR_010840 | A2067D | 0.40 | 1.632 | -0.32 | III |  | [5] |
| VAR_010841 | V2079I | 0.23 | 0.089 | -0.72 | II |  | [14] |
| VAR_010842 | E2139G | 0.15 | 2.284 | -1.54 | II |  | [19] |
| VAR_010843 | E2164K | 0.53 | 1.609 | -0.82 | II |  | [15] |
| VAR_010844 | S2218C | 0.00 | 1.544 | -0.46 | II |  | [20] |
| VAR_010846 | R2227C | 0.56 | 2.654 | -0.72 | II |  | [5] |
| VAR_010848 | A2274T | 0.46 | 1.542 | -0.72 | II |  | [13] |
| VAR_010849 | G2287A | 0.18 | 1.613 | -0.8 | II |  | [14] |
| VAR_041573 | L2307S | 0.48 | 1.568 | -0.7 | II |  | [7] |
| VAR_041574 | L2332P | 0.00 | 0.473 | -1.52 | II |  | [7 |
| VAR_056689 | T2335K | 0.00 | 1.47 | -1.14 | II |  |  |
| VAR_041575 | I2356F | 0.08 | 1.799 | -1.22 | II |  | [7] |
| VAR_010850 | T2396S | 0.00 | 1.059 | -0.96 | II |  | [15] |
| VAR_041576 | S2408L | 0.00 | 2.149 | 0.41 | II |  | [7] |
| VAR_010852 | A2420P | 0.00 | 1.767 | -0.34 | II |  | [21] |
| rs28942102 | E2422G | 0.23 | 2.284 | -0.72 | II | -NA- |  |
| **VAR_010853** | **E2423G** | **0.04** | **2.284** | **-0.77** | **I** |  | [18] |
| **VAR_010854** | **V2424G** | **0.00** | **2.33** | **-1.89** | **I** |  | [15] |
| **VAR_010856** | **T2438I** | **0.05** | **2.05** | **-0.77** | **I** |  | [11] |
| VAR_010857 | Q2442P | 1.00 | 2.121 | -0.45 | III |  | [7] |
| VAR_041577 | R2443Q | 0.07 | 1.558 | -0.81 | II |  | [7] |
| VAR_041578 | C2464R | 0.73 | 3.223 | -0.48 | III |  | [7] |
| VAR_010858 | Y2470D | 0.6O | 2.983 | -1.24 | II |  | [5] |
| VAR_010859 | R2486G | 0.68 | 2.429 | -1.25 | II |  | [22] |
| VAR_010860 | W2491R | 0.13 | 3.902 | -1.06 | II |  | [23] |
| VAR_041579 | L2492R | 0.29 | 2.018 | -1.56 | II |  | [7] |
| rs35203200 | V2540I | 0.56 | 0.98 | -0.99 | III | ESE |  |
| VAR_010862 | H2554D | 0.16 | 2.921 | -0.27 | III |  | [10] |
| **VAR_056690** | **E2570G** | **0.03** | **1.731** | **-1.23** | **I** |  |  |
| **VAR_010863** | **D2625Q** | **0.01** | **1.731** | **-0.53** | **I** |  | [11] |
| VAR_056691 | T2640I | 0.08 | 1.788 | -0.33 | III |  |  |
| VAR_010865 | L2656P | 0.06 | 2.051 | -1.65 | II |  | [24] |
| rs34099398 | M2667V | 0.48 | 1.984 | -0.74 | II | ESE |  |
| VAR_010868 | E266G | 0.54 | 2.009 | -1.08 | II |  | [10] |
| **rs28942103** | **Y2677C** | **0.00** | **2.758** | **-0.5** | **I** | -NA- |  |
| VAR_010869 | G2695A | 0.59 | 1.824 | -1.05 | II |  | [13] |
| VAR_010870 | I2702R | 0.53 | 2.474 | -1.59 | II |  |  |
| VAR_056692 | G2709S | 0.49 | 1.824 | -1.47 | II |  |  |
| VAR_041581 | R2719H | 0.39 | 1.551 | -1.29 | II |  | [7] |
| VAR_010871 | L2722H | 0.30 | 2.018 | -1.5 | II |  | [15] |
| VAR_010872 | D2725G | 0.31 | 2.189 | -0.1 | III |  | [25] |
| VAR_010873 | D2725V | 0.95 | 2.639 | -0.86 | II |  | [15] |
| VAR_010874 | A2726V | 0.02 | 1.542 | -0.34 | II |  |  |
| VAR_010875 | F2732L | 0.06 | 2.153 | -0.74 | II |  | [15] |
| VAR_010876 | G2765S | 0.36 | 1.824 | -1.13 | II |  | [7] |
| VAR_010878 | C2824Y | 0.14 | 3.346 | -0.3 | III |  | [27] |
| VAR_010879 | F2827C | 0.13 | 2.603 | -1.66 | II |  | [10] |
| VAR_010880 | P2829L | 0.12 | 2.846 | -0.26 | II |  | [9] |
| VAR_010881 | R2832C | 0.90 | 2.654 | -0.97 | II |  | [11] |
| **VAR_041582** | **P2842R** | **0.00** | **2.621** | **-0.75** | **I** |  | [7] |
| VAR_010882 | R2849P | 0.22 | 2.429 | -0.57 | II |  | [5] |
| VAR_010883 | S2855R | 0.92 | 1.924 | -0.26 | III |  |  |
| **VAR_010886** | **G2867R** | **0.05** | **2.274** | **-0.65** | **I** |  | [5] |
| VAR_041583 | D2870N | 1.00 | 1.739 | -1.19 | II |  | [7] |
| VAR_010888 | L2890V | 0.02 | 1.343 | -1.6 | II |  | [15] |
| VAR_010889 | R2904G | 1.00 | 2.284 | -1.25 | II |  | [26] |
| **rs56887719** | **P2907L** | **0.00** | **2.846** | **-0.66** | **I** | -NA- |  |
| **VAR_010890** | **E2909G** | **0.01** | **2.429** | **-1.4** | **I** |  | [23] |
| **rs1137889** | **N3003D** | **0.04** | **1.722** | **-0.57** | **I** | -NA- |  |
| VAR_010892 | A3006P | 0.66 | 1.603 | -0.54 | II |  | [25] |
| VAR_010893 | R3008C | 0.56 | 2.135 | -0.98 | II |  | [11] |
| VAR_010894 | R3008H | 0.71 | 1.868 | -1.47 | II |  | [21] |
| VAR_010895 | K3018N | 0.53 | 1.843 | -0.2 | II |  | [21] |
